# Mass cytometric analysis of the immune cell landscape after traumatic brain injury elucidates the role of complement and complement receptors in neurologic outcomes

**DOI:** 10.1186/s40478-023-01583-0

**Published:** 2023-06-12

**Authors:** Amer Toutonji, Carsten Krieg, Davis M. Borucki, Mamatha Mandava, Silvia Guglietta, Stephen Tomlinson

**Affiliations:** 1 College of Medicine, Medical University of South Carolina, Charleston, SC 29425 USA; 2 Department of Pathology and Laboratory Medicine, Medical University of South Carolina, Charleston, SC 29425 USA; 3 Hollings Cancer Center, Charleston, SC 29425 USA; 4 Department of Microbiology and Immunology, Medical University of South Carolina, Charleston, SC 29425 USA; 5 Immune Deficiency Cellular Therapy Program (IDCTP), National Institutes of Health, Bethesda, USA; 6 Department of Regenerative Medicine and Cell Biology, Medical University of South Carolina, Charleston, SC 29425 USA; 7 Ralph Johnson VA Medical Center, Charleston, SC 29401 USA

**Keywords:** Traumatic brain injury, Complement, Neuroinflammation, Complement inhibition, Microglia, Mass cytometry

## Abstract

Following traumatic brain injury (TBI), a neuroinflammatory response can persist for years and contribute to the development of chronic neurological manifestations. Complement plays a central role in post-TBI neuroinflammation, and C3 opsonins and the anaphylatoxins (C3a and C5a) have been implicated in promoting secondary injury. We used single cell mass cytometry to characterize the immune cell landscape of the brain at different time points after TBI. To specifically investigate how complement shapes the post-TBI immune cell landscape, we analyzed TBI brains in the context of CR2-Crry treatment, an inhibitor of C3 activation. We analyzed 13 immune cell types, including peripheral and brain resident cells, and assessed expression of various receptors. TBI modulated the expression of phagocytic and complement receptors on both brain resident and infiltrating peripheral immune cells, and distinct functional clusters were identified within same cell populations that emerge at different phases after TBI. In particular, a CD11c+ (CR4) microglia subpopulation continued to expand over 28 days after injury, and was the only receptor to show continuous increase over time. Complement inhibition affected the abundance of brain resident immune cells in the injured hemisphere and impacted the expression of functional receptors on infiltrating cells. A role for C5a has also been indicated in models of brain injury, and we found significant upregulation of C5aR1 on many immune cell types after TBI. However, we demonstrated experimentally that while C5aR1 is involved in the infiltration of peripheral immune cells into the brain after injury, it does not alone affect histological or behavioral outcomes. However, CR2-Crry improved post-TBI outcomes and reduced resident immune cell populations, as well as complement and phagocytic receptor expression, indicating that its neuroprotective effects are mediated upstream of C5a generation, likely via modulating C3 opsonization and complement receptor expression.

**Supplementary Information:**

The online version contains supplementary material available at 10.1186/s40478-023-01583-0.

## Introduction

Traumatic brain injury (TBI) is a leading cause of mortality and chronic disability, both in the civilian and military population, with a considerable socio-economic impact. Following the primary injury, a secondary injury develops which involves a chronic neuroimmune process that can last years. Notably, secondary neuroinflammation following TBI is an influential factor in the development of neurodegenerative pathologies such as Alzheimer’s disease and Parkinson’s disease, as well as chronic traumatic encephalopathies [1, 2]. To date, available interventions post TBI focus on prevention of brain herniation acutely and symptomatic management of neuropsychiatric manifestations chronically, but these interventions do not protect against ongoing neuroinflammation [3].

We and others have shown that the complement system plays an important role in TBI [4–8]. Of note, while inhibition of complement either early or late in the pathway is neuroprotective in the acute phase after TBI, late (terminal) pathway inhibition fails to provide protection from the chronic sequalae of TBI in an experimental model. These findings suggest that complement-mediated pathology following TBI may be associated with the ability of complement opsonins and anaphylatoxins to recruit and activate resident and peripheral immune cells, which can exacerbate brain damage. In this context, we have documented an aberrant process of microglial phagocytosis of complement opsonized neurons and synapses following murine TBI [7, 9].

Several studies have indicated that infiltration of peripheral immune cells and activation of resident mononuclear phagocytes occurs during TBI and contributes to neuroinflammation and secondary injury [10–12]. However, anti-inflammatory drugs have not only failed to show clinical benefit, but in some instances have had deleterious effects [13]. This may be because the inflammatory process following TBI has dual roles in promoting neuronal death and neurodegeneration on the one hand, and facilitating neuronal repair and clearance of debris and dead cells on the other [14–16]. For example, there may be multiple subsets of the same immune cell populations with different time-dependent functions and effects on recovery. Therefore, a better understanding of temporally regulated cellular and molecular mechanisms of the inflammatory response following TBI and discriminating between the processes associated with the neurodegenerative sequalae of TBI vs. the processes that can support repair and regeneration, will be essential for the development of more effective TBI treatments that can reduce brain tissue damage and promote neuronal survival, repair, and regeneration.

In this study we used single cell mass cytometry (CyTOF) as an unbiased and comprehensive approach to characterize the immune cell landscape of the TBI brain at acute and sub-acute time points. We identified 13 immune cell types, including peripheral and brain resident immune cells, and assessed expression of various complement and phagocytic receptors on these cells. Furthermore, to understand the effects of complement activation on immune cell infiltration and immune-cell mediated phagocytosis, we used an injury site-targeted inhibitor of C3 activation, CR2-Crry, which has been shown to confer neuroprotection when administered at either acute or chronic time points after TBI [7, 9, 17]. The CR2 component binds to complement C3 activation products deposited at the site of injury, hence acting as an injury site-targeting moiety. The Crry component inhibits C3 activation and prevents the production of complement effector molecules by all complement activation pathways. Based on our analysis of immune cell receptors, we also used PMX-205, a C5aR1 antagonist, to investigate the role of C5a anaphylatoxin in cellular inflammation and neurological recovery.

## Materials and methods

### Animals

C57BL/6 wild type (WT) mice were purchased from Jackson laboratories and maintained in a SPF (Specific Pathogen Free) animal facility at the Medical University of South Carolina. Mice had access to regular chow food and water ad libitum and were housed on a 12-h day-night cycle in laminar flow racks in a temperature-controlled room (25 °C). Male mice were used throughout the study. All experiments were performed during the light cycle. The animals were housed in individually ventilated cages with corn cob bedding. All animal experiments were performed under protocols approved by the Institutional Animal Care and Use Committee at MUSC and Ralph H. Johnson VA Medical Center.

### Controlled cortical impact injury

Controlled cortical impact (CCI) was performed as previously described [17]. Briefly, 12-week-old male C57BL/6 mice were anaesthetized with ketamine (100 mg/kg i.p.) and xylazine (10 mg/kg i.p.). The scalp was then cut longitudinally in the middle, the skin retracted, and a 4 mm diameter hole drilled halfway between the lambda and bregma and 0.5 mm to the right of the midline (parasagittal) above the right sensorimotor cortex. The exposed intact dura was then hit with a pneumatic impactor device (Infinite Horizon, Precision Scientific Inc.) at a depth of 2.5 mm, a velocity of 6 m/s, a dwell time of 100 ms, and a 10° angle relative to vertical axis. The scalp incision was then closed using clips and animals allowed to recover on warmed bedding with access to soft food and water before being returned to their home cages. Sham mice received only anesthetic and a scalp incision without craniotomy.

### Magnetic resonance imaging (MRI)

Lesion volume was quantified by MRI scans, which were acquired on day 27 after TBI using a 7 T/30 Bruker BioSpec animal scanner (Billerica, MA). T2-weighted images, which clearly separated the CSF from the lesion at chronic time points, were used to quantify lesion size by measuring the volume of T2-hypointensities from 15 equidistant 2-mm thick sections. Free-access software Horos was used to trace the lesions in consecutive sections and compute a 3D volume in mm^3^.

### Complement inhibitors

The preparation and purification of the recombinant fusion protein CR2-Crry have been previously described [18]. Protein purity was assessed by SDS-PAGE, and complement inhibitory activity was evaluated by zymosan assay according to previously published protocols [18, 19]. All preparations were confirmed to be endotoxin free before injection. For complement inhibition, mice received CR2-Crry (16 mg/Kg) via intravenous injection 1-h post TBI, and mice kept for 28 days post-injury received three additional doses at the beginning of weeks 2, 3 and 4 after injury to ensure sustained complement inhibition. Control mice were treated with PBS vehicle. The C5aR1 antagonist, PMX205, was purchased from Tocris Bioscience and administered by sub-cutaneous injection at a dose of 1 mg/Kg one hour post TBI and every other day for the duration of the experiment. A recent publication studying the pharmacokinetics and pharmacodynamics of PMX205 has shown that subcutaneous administration results in the highest bioavailability in the brain and that PMX205 levels remain above IC50 required for the inhibition of C5aR1 on immune cells in vitro for 5 days after a single 1 mg/kg intravenous injection [20, 21].

### Preparation of single cell suspensions from the brain

Brain single cell suspensions were prepared for flow cytometry and mass cytometry using previously published protocols with minor modifications [22, 23]. Briefly, mice were euthanized with an overdose of isoflurane and transcardially perfused with 15 ml cold PBS. The ipsilateral brain hemispheres and meninges were gently minced using a scalpel and digested in Hibernate-A medium with 0.4 mg/ml Collagenase D (Sigma-Aldrich) for 30 min at 37 °C on a magnetic stirrer. Cell suspensions were then filtered, and myelin and debris eliminated by Percoll gradient centrifugation for 30 min at 4 °C. Cells were finally washed in cold PBS and counted.

### Mass cytometry

Mass cytometry antibodies were either labelled in-house using antibody-labelling kits and protocols according to manufacturer’s instructions (Fluidigm) or purchased from Fluidigm. Prior to labelling, antibodies were individually titrated and optimized for concentration using flow cytometry before use in the final mass cytometry panel. For live cell barcoding, we followed the protocol by Mei et al. using six palladium metal isotopes for live-cell barcoding of samples with CD45.2 [24]. The barcodes used in this study consisted of unique combinations of 3 out of 6 antibody-heavy metal complexes, yielding 20 possible barcodes (Additional file 1: Table S1). Briefly, 1 × 10^6^ cells from individual samples were incubated with respective CD45-Pd antibodies in cell staining buffer at 4 °C, washed and combined into one composite sample. To identify dead cells, 2.5 μM cisplatin in PBS was added for 2 min at RT. The composite sample was stained with a cocktail of 33 antibodies (Table 1), washed and fixed with 2% paraformaldehyde (PFA; Electron Microscopy Sciences) at 4 °C. Fixed cells were then incubated with DNA-intercalating solution (Iridium (Sigma) in MaxPar Fix/Perm buffer (Fluidigm) overnight at 4 °C. Before acquisition, samples were washed twice with MilliQ water. Barcoded composite samples were acquired on a Helios mass cytometer (Fluidigm). Quality control and tuning processes on the Helios was performed daily before acquisition. Data from different days and across acquisition time was normalized by adding five-element beads to the sample immediately before acquisition and using MATLAB-based normalization software, as described previously [25, 26]

**Table 1:** List of CyTOF antibodies and their conjugated heavy metals

#	Markers	Company	Clone	Catalog #	Metal	Isotope
1	Axl	R&D	175,128	FAB8541G	Yb	171
2	B220	Biolegend	RA3-6B2	103,249	Nd	144
3	BAI-1	Novus Biol	Polyclonal	NB110-81,586	Yb	173
4	C3aR	Hycult	14D4	HM1123	Dy	161
5	C5aR1	Biolegend	20/70	135,806	Gd	160
6	C5aR2	R&D	468,705	MAB4729	Nd	145
7	CCR2	R&D	475,301	MAB55381	Dy	163
8	CD117	Biolegend	2B8	105,829	Er	166
9	CD11b	Biolegend	M1/70	101,249	Nd	148
10	CD11c	Biolegend	N418	117,341	Bi	209
11	CD16/32	Biolegend	93	101,335	Er	167
12	CD163	Biolegend	S15049I	155,302	Er	170
13	CD206	Biolegend	C068C2	141,702	Er	168
14	CD3	Biolegend	145-2C11	100,345	Sm	152
15	CD36	Biolegend	HM36	102,602	Nd	146
16	CD45	Biolegend	30-F11	103,102	Sm	147
17	CD64	Biolegend	X54-5/7.1	139,302	Eu	151
18	CD68	Biolegend	FA-11	137,002	Sm	149
19	CX3CR1	Biolegend	SA011F11	149,007	Dy	164
20	F4/80	Biolegend	BM8	123,143	Tb	159
21	LRP1	Thermofisher	A2MR-beta-1	37–7600	Gd	155
22	Ly6C	Biolegend	HK1.4	128,039	Nd	150
23	Ly6G	Biolegend	1A8	127,637	Pr	141
24	MerTK	Biolegend	2B10C42	151,502	Nd	143
25	MHCII	Biolegend	M5/114.15.2	107,637	Yb	174
26	NK1.1	Biolegend	PK136	108,743	Ho	165
27	P2XR7R	Biolegend	1F11	148,702	Nd	142
28	P2Y12R	Biolegend	S16007D	848,002	Dy	162
29	SCARA1	Biolegend	1F8C33	154,702	Lu	175
30	SCARB1	Novus Biol	Polyclonal	NB400-101	Eu	153
31	Siglec H	Biolegend	551	129,602	Gd	158
32	SIRPa	Biolegend	P84	144,002	Sm	154
33	VNR	Biolegend	2C9.G2	104,310	Yb	172

### Mass cytometry data analysis

Mass cytometry data were analyzed following a previously published workflow [26]. Briefly, after acquisition, the composite sample was de-barcoded using FlowJo software, a diagnostic analysis was performed to ensure successful tissue processing and staining with all antibodies, and a dimensionality reduction plot of all samples based on the expression of all markers was generated to identify potential outliers. Subsequently, all samples were imported in R and analyzed using a previously published code based on the CATALYST and diffcyt packages [27]. Additional data visualization utilized the *tidyverse* suite of packages extensively explained in R for Data Science by Hadley Wickham. Of note, per sample, the total cell counts for the individual cell types were determined by multiplying the number of cells in a cell type by the total number of cells extracted from the injured brain, and then dividing by the total number of cells injected into the cytometer (which was 62,000 for all the samples).

### Flow cytometry

Single cell suspensions from brains were stained with a cocktail of antibodies including CD45.2 (clone 104) and CD11b (clone M1/70) from BD Biosciences along with a live/dead fixable marker (Molecular Probes). After washing and fixation in 1% PFA, data were acquired on a BD Fortessa X-20 Analytic flow cytometer and analyzed using FlowJo V10 analysis software (FlowJo, OR).

### Neurological severity score (NSS)

NSS assesses body symmetry of mice after unilateral brain injuries and ranges between 0 (normal) and 4 (worst) [28]. The scores are as follows: 0—can walk in a straight line and can easily tilt in both directions easily when lifted by the tail, 1—can walk in a straight line but only tilts to one side when lifted by the tail, 2—can walk in a straight line but circles around body axis or doesn’t tilt to either side when lifted by the tail, 3—walks in circles only, 4—can’t move at all.

### Open field ambulation (OFA)

Mice were placed in a 40 cm × 40 cm white plastic box and allowed to move around freely for 15 min while being video recorded using Noldus^®^ EthoVision software. The distance walked between 3 and 15 min was quantified in an automated manner to assess physical activity. The first 3 min were discarded to control for anxiety caused by animal handling and being in a new or foreign setting. OFA was assessed before and after injury for normalization of post-injury activity by pre-injury activity.

### Barnes maze (BM) and reverse Barnes maze (reverse BM)

To assess spatial memory following TBI, we used Barnes maze, which tests the ability of mice to find an escape hole relative to the position of visual cues. Mice were trained over 5 consecutive days, with two 5-min trials per day separated by 1–2 h, to find the escape hole. The training started 14 days after injury in the case of the mice used for the CyTOF experiment and day 7 after injury in the PMX205 experiment. The training was followed by a retention session two days after last training. Each session was recorded using EthoVision XT software, and the distance and time taken to find the escape hole on each trial was computed in an automated manner. Reverse BM was performed on day 21 post injury on the animals treated with C5aR antagonist using the same schedule of BM (5 training days and 1 retention day). In the case of reverse BM, we repositioned the escape hole at a 120° angle relative to its position in BM.

### Statistical analysis

Statistical analyses for behavioral tasks, lesion volume, and flow cytometry were performed using GraphPad Prism 9.0 (GraphPad, CA). Parametric testing was used for all outcome assessments except NSS. Group analyses were performed using one-way ANOVA followed by multiple comparison analyses (e.g., immune cell numbers in naïve vs PBS vs PMX205 groups) or using repeated measures ANOVA for matched data (e.g., behavioral outcomes for the same mice from naïve vs PBS vs CR2-Crry groups at several time points). *p*-values below 0.05 were considered significant and were adjusted using Bonferroni correction unless otherwise specified. Student’s t-test was used to compare two groups and was always used as two-tailed. Data is reported as Mean ± SEM unless otherwise specified. Sample size was calculated using G*Power 3 (Universität Düsseldorf, Germany) based on an effect size determined for each outcome measure by previous studies and using a power ≥ 80%. Surgeons and investigators were blind to experimental groups, and behavioral assessments were scored using automated Noldus^®^ EthoVision software. Experimental groups were coded and were only revealed after the final quantification of outcome measures. Statistical analysis for mass cytometry data was done using CATALYST and diffcyt packages in R. The percentages of various immune cell types under different experimental conditions were compared using a generalized linear mixed model (GLMM) that adjusted for potential differences between batches. The levels of expression of various receptors by various immune cell types under different experimental conditions were compared using a linear mixed model (LMM), which also adjusted for potential batch effects. False discovery rate was used to adjust for the number of comparisons and was considered significant when below 0.05.

## Results

### Temporal analysis of resident and infiltrating peripheral immune cells in the brain after TBI

Studies in preclinical models, as well as histological examination of specimens from patients, have shown that TBI results in the recruitment and activation of resident and circulating immune cells, both of which have been implicated in promoting secondary injury, neuronal loss and neurodegeneration. The complement system has also been implicated in these secondary mechanisms after TBI, and in this study we performed a detailed characterization the cellular immune phenotype of the post-TBI brain in the context of complement activation and inhibition.

**Fig. 1:**
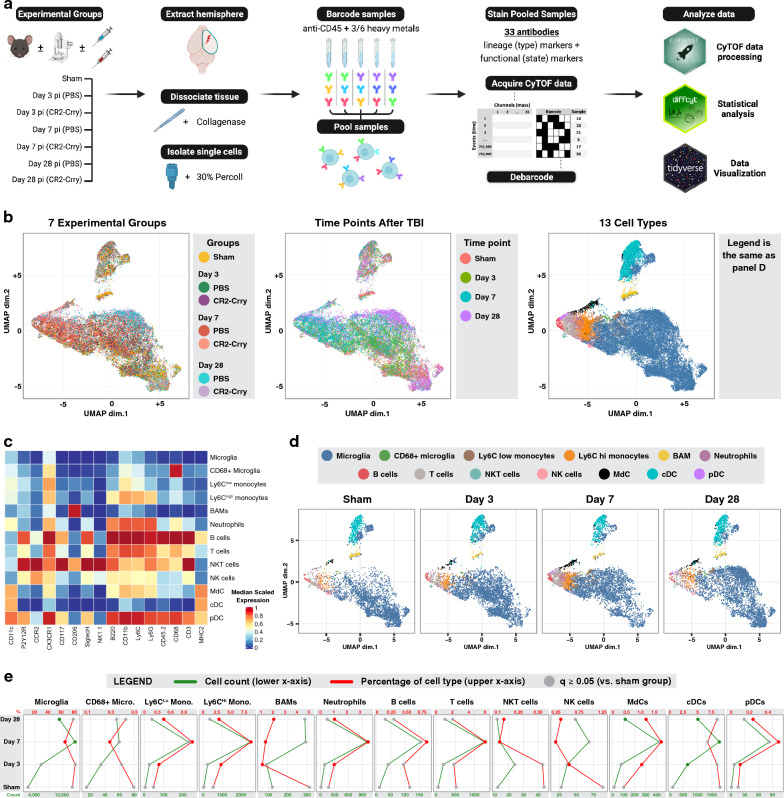
Temporal analysis of resident and infiltrating peripheral immune cells in the brain after TBI. **A** Graphical workflow of the mass cytometry experiment including the isolation of immune cells from the brains of 7 experimental groups, the staining and processing of the cells, and the R packages used to analyze the data. **B** A lineage UMAP showing a total of 42,000 cells (1000 per sample) plotted based on the expression of lineage markers. Four versions are shown color-coded respectively by the experimental group, the time point after traumatic brain injury, the 40 lineage clusters returned by the FlowSOM algorithm, and the 13 immune cell types derived from the manual annotation of the 40 lineage clusters (see also Additional file 1: Fig. S1). **C** Heatmap showing lineage marker expression of the 13 identified cell types. **D** Lineage UMAP depicting cells from the sham and PBS-treated groups at days 3, 7, and 28 in separate subpanels, color-coded by the immune cell type. **E** Quantification of the cell count (green) and the percentage (red) of each of the 13 immune cell types in the sham and PBS-treated groups at days 3, 7, and 28. Data are represented as mean. Significant changes compared to the sham group are in green and red dots; non-significant changes are in gray. N = 6 per group. False discovery rate was used to adjust the *p*-value. Abbreviations: cDC = conventional dendritic cell, pDC = plasmacytoid dendritic cell, MdC = monocyte-derived cell

Following TBI by controlled cortical impact (CCI), mice were randomized into groups and treated with either CR2-Crry or PBS (vehicle). A control sham group was also included. Groups of mice were euthanized on day 3, 7 or 28 post-TBI, and brain-derived single cell suspensions from injured hemispheres were barcoded using a 6-choose-3 anti-CD45 barcoding scheme (Additional file 1: Table S1). Samples were then pooled and stained with a cocktail of 33 antibodies that included lineage markers as well as functional markers that included several phagocytic and complement receptors (Table 1). This was done with three batches for which surgeries and tissue processing were done on consecutive days. Each batch included two samples from each group (hence, total N of 6 per group). Following the preparation of all batches, data from a minimum of 1 × 10^6^ cells/batch was acquired by mass cytometry, de-barcoded, and analyzed as shown in the summary workflow in Fig. 1a.

Based on the differential expression of 16 lineage markers, we generated UMAPs using 1000 single cells from each of the 42 experimental samples (6 mice for each time point: 3, 7 and 28 days, with and without CR2-Crry treatment, and 6 mice in the sham group). As shown in Fig. 1b, when UMAPs were coded based on experimental condition and time point, they showed differential clustering by color, indicating that immune cells from the different experimental groups and time points have significant differences in expression of lineage markers indicating different cell types. We next examined sham and vehicle groups and used a third UMAP to represent the distribution of 40 cell clusters generated using the FlowSOM algorithm (Additional file 1: Fig. S1). These 40 clusters were subsequently manually annotated and merged into 13 cell clusters guided by lineage marker expression as described [22] and as shown in the heatmap in Fig. 1c. Comparison of the UMAPs from sham mice and injured mice at different time points post-TBI (Fig. 1d) clearly shows differences in the densities of various cell types (e.g., increased density of Ly6C^high^ monocytes shown in orange at day 7), along with differential clustering within microglia. We next quantified the percentage and total numbers of each cell type in sham and in TBI mice; while microglia represented the most abundant immune cell type in all conditions, their percentage, as well as the percentage of border-associated macrophages (BAMs), showed a significant decrease at day 7 post-TBI compared to sham (Fig. 1e). Meanwhile, the percentage of most types of peripheral immune cell was increased in TBI mice compared to sham at this time point, and was most pronounced for Ly6C^high^ monocytes, neutrophils, and T cells. However, at day 28 post-TBI, the percentage of microglia relative to other immune cell types increased to 75% after a decrease of 62% seen at day 7 post-TBI. Moreover, the overall number of microglia remained elevated at day 28 compared to sham brains.

### TBI modulates the expression of phagocytic and complement receptors on both brain resident and infiltrating peripheral immune cells

Following the phenotypic characterization of immune cells in the brain after TBI, we investigated how the observed changes in the expression of functional markers on immune cells were associated with a post-TBI neuroinflammatory response. On each of the 13 immune cell types, we examined the expression of 26 functional markers, including 5 complement receptors and 21 other phagocytic, chemotactic, and purinergic receptors. To note, it has been shown that complement C3 opsonins can mediate the phagocytosis of viable perilesional neurons and synapses after TBI [17].

**Fig. 2:**
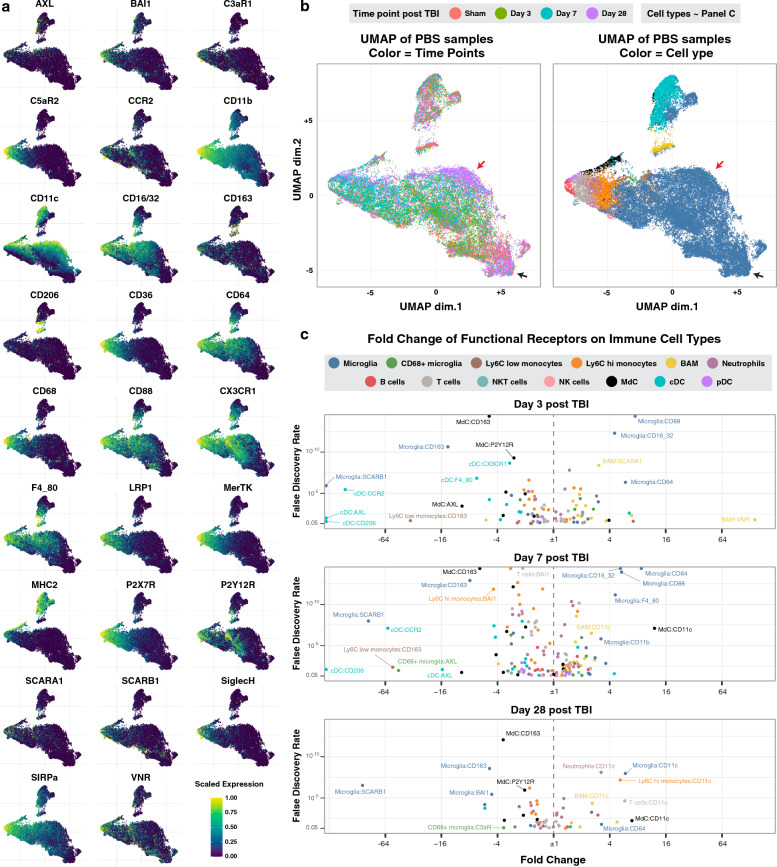
TBI modulates the expression of phagocytic and complement receptors on brain resident and infiltrating peripheral immune cells. **A** Lineage UMAPs of sham and PBS-treated experimental groups showing the relative expression of 26 functional markers. **B** The same lineage UMAPs color-coded by time point after TBI and immune cell types. Overlay visually with the UMAPs in panel A for an easier understanding of the dynamic expression of the functional markers by time point and cell type. **C** Volcano plots quantifying the fold change in immune cell expression of the functional markers at all time points in the PBS-treated group compared to the sham group (see also Additional file 1: Fig. S2). Each data point represents the mean in fold change for a receptor-cell type combination. N = 6 per group. False discovery rate was used to adjust the *p*-value. Abbreviations: cDC = conventional dendritic cell, pDC = plasmacytoid dendritic cell, MdC = monocyte-derived cell

Figure 2a shows the scaled expression of the 26 functional markers, each on a separate lineage based UMAP. The lighter the color is, the higher the expression of the functional marker. The same UMAPs are plotted in Fig. 2b and are color-coded by the time point after TBI and the cell type. By inspecting both panels side-by-side, it is possible to assess the expression of functional markers by each cell type at different time points after TBI. For instance, the black arrows in Fig. 2b point to a microglial subpopulation that is present in the sham brain and at day-28 after injury; this subpopulation shows low expression of all the functional markers (Fig. 2a, darker color), suggestive of surveying microglia. Meanwhile, the red arrows (Fig. 2b) point to a microglial population that is mostly present on day-28 after injury and that expresses high levels of CD11c and CX3CR1.

We next quantified the expression of functional markers on all cell types at 3, 7 and 28 days after TBI, as well as in sham animals (Fig. 2c, Additional file 1: Fig. S2). On days 3 and 7 post-TBI, CD88 (C5aR1) and the IgG receptors CD16/32 and CD64 were significantly upregulated on most immune cells and in particular on brain resident microglia and border associated macrophages (BAMs). Border associated macrophages also showed an up-regulation of the scavenger receptors VNR and SCARA-1 (Fig. 2c); these particular receptors were not upregulated on infiltrating cell types such as Ly6C^high^ monocytes and neutrophils.Conversely, the scavenger receptors CD163 and SCARB1 were significantly downregulated on microglia, as well as on other immune cells including BAM, Ly6C^high^ and Ly6C^low^ monocytes, neutrophils, T cells and dendritic cells (Additional file 1: Fig. S2). To note, CD163 is a high affinity scavenger receptor for the hemoglobin-haptoglobin complex and may be specifically downregulated after hemorrhagic trauma because of the uptake of hemoglobin released from lysed red blood cells. Interestingly, at 28 days post-TBI, both brain-resident and infiltrating peripheral mononuclear phagocytes showed a significant up-regulation of complement receptor 4 (CR4, CD11c/CD18). Together, the above data suggest that while there is significant infiltration and priming of peripheral immune cells acutely after TBI, resident immune cells undergo more pronounced changes in the expression of functional markers, and resident immune cells appear to be in a higher primed state for phagocytic activity compared to infiltrating immune cells.

### The expression of functional markers identifies distinct functional clusters within same cell populations that emerge at different phases after TBI

**Fig. 3:**
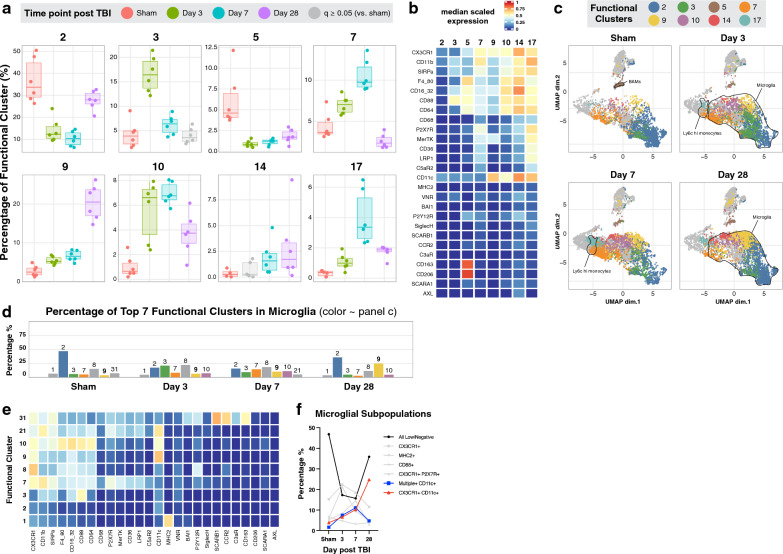
The expression of functional markers identifies distinct subpopulations of immune cells that emerge at different phases after TBI. **A** FlowSOM clustering based on the expression of functional markers identified 40 functional clusters (see also Additional file 1: Fig. S3). In this panel, 8 functional clusters with high differential abundance at different time points are shown here. **B** Heatmap of the 8 functional clusters showing their median scaled expression of 26 functional markers. **C** Lineage UMAP color-coded by the 8 functional clusters to show their distribution amongst the 13 immune cell types. The percentage **D** and heatmap **E** (same scale as panel B) of the 7 most abundant microglial functional clusters per time point. **F** Summary of the abundance of distinct microglial functional clusters labeled by the most highly expressed functional markers. Data are shown as mean in panels D and F. **A–F** N = 6 per group. False discovery rate was used to adjust the *p*-value in panel A. Abbreviations: cDC = conventional dendritic cell, pDC = plasmacytoid dendritic cell, MdC = monocyte-derived cell

Despite strong evidence that neuroinflammation plays an important role in secondary injury after TBI, anti-inflammatory drugs administered post-TBI have failed to prevent neurodegeneration in clinical trials, and in some cases have worsened outcomes [13]. A possible explanation for this is that while some components of the inflammatory process may be detrimental, others may be important for protection and recovery processes in the brain. As such, the function rather than the phenotype of defined cell populations may dictate a protective vs. injurious role in the neuroinflammatory process following brain injury. Therefore, we investigated the emergence of distinct functional profiles and their abundance after TBI using the FlowSOM algorithm to cluster all cells in the dataset into 40 functional clusters based on the expression of 26 functional markers (Additional file 1: Fig. S3). As shown in Fig. 3a, b, among the 40 functional clusters identified, clusters 2, 3, 5, 7, 9, 10, 14, and 17 showed the greatest differential abundance at specific time points. When these subsets of functional clusters were projected on the lineage based UMAP to match them with cell types, most of the clusters were identified as microglia, suggesting the presence of dynamic microglial subpopulations (Fig. 3c).

Cluster 5, which expressed high levels of CD163 and CD206, and intermediate levels of C5aR1 and the Fc receptors CD64 and CD16/32 (Fig. 3b), consisted primarily of BAMs (Fig. 3c). This cluster contributed to 4–12% of immune cells in the uninjured brain and decreased to 1–2% acutely after injury (Fig. 3a). Clusters 7 and 17, which showed peak abundance acutely after TBI, represented two functional clusters of peripheral Ly6C^high^ monocytes and microglia (Fig. 3c). Specifically, cluster 7 expressed CD11b (complement receptor 3, CR3), and cluster 17 expressed several functional receptors, with CR3 and CR4 among the receptors with the highest level of expression.

Based on these findings, we focused on the most abundant functional clusters within microglia at different time points following TBI. Interestingly, as shown in Fig. 3d–f, cluster 2 represented almost half of the microglia in the uninjured brain and decreased following injury with only partial return to baseline at day 28. In terms of functional markers, cluster 2 was characterized by low expression of all functional markers (Fig. 3e), likely indicating a resting microglia subset. Cluster 8, characterized by high expression of CX3CR1 and intermediate to low expression of C5aR1, Fc and purinergic receptors, made up around 15% of microglia in sham brains with little change after injury. Clusters 3, 7, and 10 showed minimal abundance in the uninjured brain, peaking acutely following TBI and returning almost to baseline levels by day 28. Clusters 3 and 7 were characterized by high expression of complement receptors C5aR1 and CR3, respectively. Cluster 10 showed intermediate expression of C5aR1, CR4 and the Fc receptors CD64 and CD16/32. Finally, cluster 9, which is characterized by high expression of CR4, constituted a very small percentage of microglia in the uninjured brain but showed a continuous increase from 4% on day 3 post-TBI to about 25% of microglia on day 28. These data together with our finding that CR4 expression is significantly up regulated on several innate and adaptive immune cells at 28 days post TBI suggest an important role for CR4 in sub-acute and chronic phases of TBI. Of note, this was the only receptor to show continuous increase in expression levels over time after TBI.

### Complement inhibition affects the abundance of brain resident immune cells in the injured hemisphere and impacts the expression of functional receptors on infiltrating cells

We have previously shown that the injury site-targeted complement inhibitor, CR2-Crry, is protective in the TBI model used here; CR2-Crry administered either acutely or up to 2 months after TBI was protective as measured by histologic and neuroinflammatory outcomes and by reversal of an otherwise ongoing cognitive decline [7, 9]. Additionally, we recently performed a longitudinal transcriptomic analysis using the NanoString platform and found that CR2-Crry administered following the same schedule as used here had a brain-global anti-inflammatory effect. Specifically, CR2-Crry treatment resulted in the reduction of several inflammatory gene transcripts and the up-regulation of genes related to neuronal and synaptic markers, suggestive of reduced neuronal death [29]. Here, we investigated the effect of complement inhibition on the phenotypic and functional profile of brain-resident and infiltrating immune cells.

**Fig. 4:**
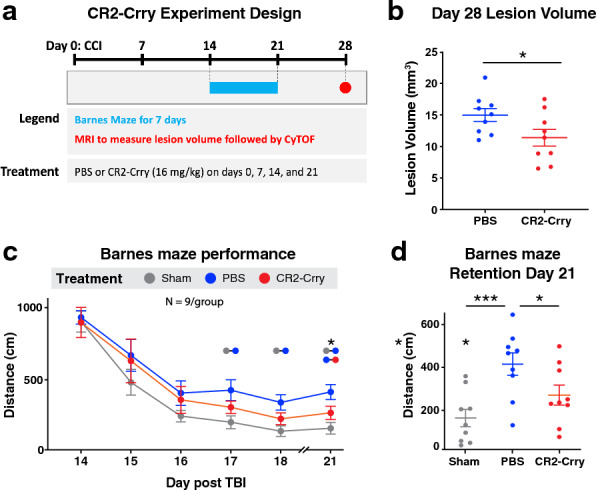
Complement inhibition improves histological and cognitive outcomes. **A** Brain lesion volume at day 28 after TBI in PBS vs CR2-Crry treated groups measured using MRI. **B** Barnes maze performance in week 3 after TBI in PBS vs CR2-Crry treated groups. **C** Performance on the retention day of the Barnes maze showing individual data points. Data are shown as mean ± SEM. N = 9 per group. Tissue samples were taken from 6 randomly chosen animals for the CyTOF analysis. Significance was calculated in A using unpaired t-test and in B-C using 1-way Anova with Bonferroni post-test (*p < 0.05, ***p < 0.001)

**Fig. 5:**
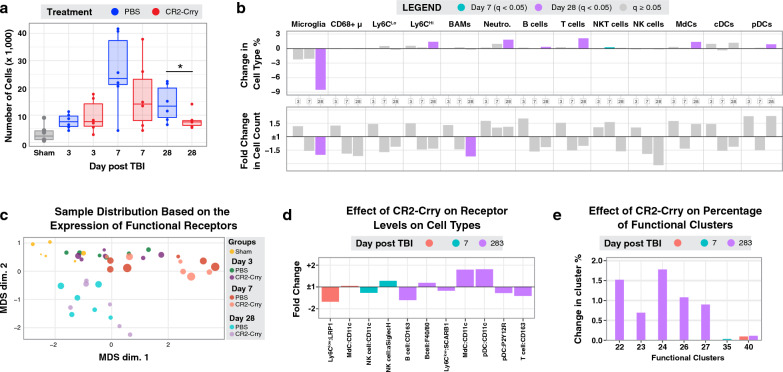
Complement inhibition affects the abundance of brain resident immune cells and the expression of functional receptors on infiltrating cells in the injured hemisphere. **A** Number of immune cells per experimental group and sample as identified by the expression of CD45 in the CyTOF panel. Data was acquired from ~ 62,000 cells from the injured hemisphere. The number of immune cells shown is normalized by the total number of isolated cells (see methods). **B** Summary of the change in percentage and the fold change in cell count of each immune cell type at days 3, 7 and 28 after TBI compared to the sham group. Non-significant changes are in gray. **C** MDS plot summarizing the differences in functional receptor expression among all 42 samples. The circle size is proportional to the number of immune cells in the sample. **D**, **E** Summary of all the significant changes in the expression of functional receptors by immune cell types **D** and percentage of functional clusters **E** upon treatment with CR2-Crry. Data are shown as mean and color-coded for statistical significance in panels B, D and E. N = 6 per group. *p < 0.05. Bonferroni correction was used for multiple comparisons in panel A. False discovery rate was used to adjust the *p*-value in the other panels. Abbreviations: cDC = conventional dendritic cell, pDC = plasmacytoid dendritic cell, MdC = monocyte-derived cell

Mice were treated with CR2-Crry or PBS and euthanized at day 3, 7 and 28 post TBI to assess immune cell profiles in the brain. For all groups, CR2-Crry treatment was administered 1 h post injury. For the 28-day group, CR2-Crry was additionally administered weekly for a total of 4 doses to achieve ongoing complement inhibition (Fig. 4a). As shown in Fig. 4b, MRI analysis revealed that CR2-Crry treatment significantly decreased lesion volume at day 28 after TBI. Furthermore, CR2-Crry significantly improved cognitive performance as measured by the Barnes maze task on day 21 after injury (Fig. 4c, d). For CyTOF analysis of phenotypic and functional markers, injured brain hemispheres were isolated at the indicated times and processed to generate single cell suspensions. While CR2-Crry treatment did not affect total cell numbers at day 3 after TBI, it did show a trend for reduction at day 7 which became significant at day 28. This phenomenon was mainly driven by a reduction in the number and frequency of brain resident immune cells (Fig. 5a). Indeed, we observed a considerable reduction in the percentage of microglia with CR2-Crry treatment. While CR2-Crry treatment caused an increase in the percentage of infiltrating immune cells, this was due to a significant decrease in the total number of microglia rather than an increase in the actual number of peripheral immune cells as shown by total cell counts (Fig. 5b). Similarly, at day 28, CR2-Crry treatment resulted in a reduced number of BAMs. These results show that complement inhibition has a major effect on resident immune cells in the injured hemisphere and suggests a role for resident immune cells in TBI outcomes that may be independent of peripheral immune cells.

Next, we investigated whether complement inhibition also affected the expression of functional receptors on resident and infiltrating immune cells. Figure 5c is a multidimensional scaling (MDS) plot showing that samples cluster separately primarily by day after injury and not by treatment group. Measured at 3 days post injury, CR2-Crry treatment resulted in the downregulation of low-density lipoprotein receptor-related protein 1 (LRP1) on Ly6C^low^ monocytes (Fig. 5d). LRP1 is a surface endocytic receptor that binds and internalizes extracellular ligands for lysosomal degradation and has recently been implicated in the development of neurodegenerative diseases [30]. CR2-Crry treatment decreased CR4 expression on NK cells on 7 day post injury, and decreased expression of P2Y12R on pDCs on day 28 post injury, which has been shown to mediate ATP-induced maturation and regulate type 1 IFN production in these cells [31]. We also observed a significant decrease in expression of the scavenger receptor SCARB-1 in Ly6C^high^ monocytes and an increase in expression of CD11c (CR4) in MdCs and pDCs, but not on microglia, at 28 days post injury.

We next analyzed how complement inhibition affected the abundance of functional clusters defined by the expression of both lineage and functional markers (Fig. 5e, Additional file 1: Fig. S4). CR2-Crry treatment resulted in a modest, but significant up-regulation of 6 functional clusters which primarily consisted of infiltrating immune cells (Fig. 5e). These included cluster 22, which made up the majority of neutrophils, and clusters 23, 24, 26, and 27, which mainly consisted of B cells, T cells, MdCs and pDCs. Interestingly, all functional clusters showing increased percentage upon treatment with CR2-Crry expressed CR4. Altogether, these data demonstrate that besides reducing the number of resident immune cells in the injured hemisphere, CR2-Crry significantly affects the expression of some phagocytic receptors on infiltrating immune cells.

### A C5aR1 antagonist reduces immune cell infiltration but has no impact on neurological outcome following TBI

**Fig. 6:**
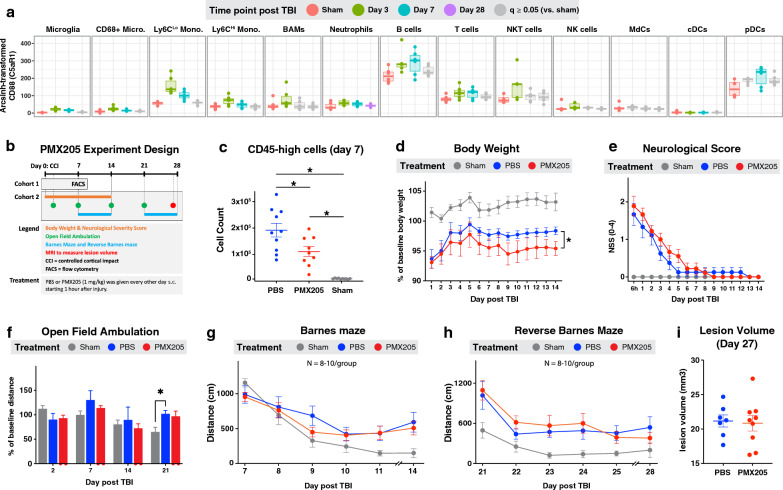
C5aR1 antagonist PMX205 reduces immune cell recruitment to injured hemisphere but has no impact on neurological outcomes following TBI. **A** Summary of C5aR1 expression by the 13 immune cell types identified by CyTOF. Non-significant changes (compared to the sham group) are in gray. **B** Graphical summary of the PMX205 experiment. Injured groups received PBS or PMX205. Sham (control) group was included. **C** Total number of CD45^high^ cells (immune cells) extracted from the injured brain hemispheres in cohort 1 and quantified by flow cytometry. N = 9–10 per group. **D**–**I** Summary of the behavioral outcomes and lesion volume over 28 days after TBI. N = 8–10 per group. **C**–**I** Data are shown as mean ± SEM. *p < 0.05. False discovery rate was used to adjust the *p*-value in panel A. Repeated measures ANOVA was used in panel D. Bonferroni correction was used for multiple comparisons in the panels. Abbreviations: cDC = conventional dendritic cell, pDC = plasmacytoid dendritic cell, MdC = monocyte-derived cell

The above data show that CR2-Crry treatment reduced the number of immune cells in the injured hemisphere following TBI and improved cognitive outcomes. These results suggest that activation of the complement system plays a role in the recruitment of immune cells that can participate in the neuroinflammatory process after TBI. In this context, the complement activation product C5a is a potent chemoattractant of immune cells to sites of complement activation and injury. The interaction of C5a with its G-protein coupled receptor, C5aR1, leads to calcium influx, activation of phosphatidylinositol 3-kinase (PI3K) and mitogen-activated protein kinases (MAPKs), and IL-6 production with consequent immune cell migration and activation [32]. As such, the C5a-C5aR1 axis has been implicated in proinflammatory signaling in several neuroinflammatory conditions, including ischemic stroke, spinal cord injury and Alzheimer’s disease [33–35]. However, a role for C5a/C5aR1 in TBI has not been investigated. We found that C5aR1 was up-regulated following TBI, and we previously reported its up-regulation at the RNA level following TBI [29]. Here, we observed a significant up-regulation of C5aR1 on 10 immune cell types at day 3 after TBI, and on 8 immune cell types at day 7 after TBI (Fig. 6a). We therefore investigated whether C5aR1 blockade affected immune infiltration and neurological outcome after TBI (Fig. 6b).

Injured mice were treated with 1 mg/kg of PMX205, a specific C5aR1 antagonist, via sub-cutaneous (s.c.) injection one hour after TBI and every other day thereafter. This dosing schedule was chosen based on reports showing that subcutaneous administration of PMX205 results in high bioavailability in the brain, and this dose effectively reduced brain amyloid deposits and glial cell reactivity in a mouse model of Alzheimer’s disease [20, 36]. Based on our data showing the highest immune cell infiltration occurred on day 7 after TBI, we euthanized one cohort of mice at this time point and performed flow cytometry. The C5aR antagonist significantly reduced the number of infiltrating mononuclear phagocytes and lymphocytes (Fig. 6c, d), confirming that C5a plays a role in recruiting peripheral immune cells to the brain after TBI. To determine the functional consequences of C5aR blockade, a second cohort of mice was treated with PMX205 and assessed 28 days after TBI. There was no difference in body symmetry evaluated by the neurological score between PMX205 and vehicle treated mice (Fig. 6e). Furthermore, PMX205 treatment did not improve functional performance as determined by open field ambulation test (Fig. 6f), or cognitive performance as determined by Barnes maze and reverse Barnes maze tasks (Fig. 6g, h). In line with these data, lesion volume was similar in PMX205 and vehicle treated mice (Fig. 6i). Together, these data show that despite reducing the number of brain-infiltrating immune cells, C5aR blockade alone does not improve outcome after TBI.

## Discussion

Despite the growing interest in understanding the neuropathology of TBI, as well as its association with chronic neuropsychiatric symptoms, there have been no extensive assessments of the immune cellular component of TBI. To date, studies have mostly focused on acute histological and flow cytometric assessments using small panels of antibodies aimed at identifying CD45^hi^ and CD45^int^ cells, a few subtypes of peripheral myeloid and lymphoid cells, and M1/M2-like myeloid phenotypes. This study provides an extensive characterization of the infiltrating and resident immune cell types in the TBI brain and their expression of 26 receptors involved in phagocytosis, chemotaxis, and complement signaling at various time points after TBI. Overall, the data showed that the number of most immune cells peaked at day 7 after TBI, with a chronically sustained increase of brain resident immune cells, namely microglia and BAMs. Moreover, early after TBI most immune cells showed upregulation of IgG receptors and complement receptor CD88, and downregulation of CD163. Notably, expression of CR4 (CD11c) increased over a 28 day period post-TBI on both infiltrating and resident immune cells, suggesting a role for this receptor in the chronic phases of the inflammatory process following TBI. In agreement with our previous transcriptomic study, microglia also expressed high levels of CX3CR1 at 28 days after TBI, suggesting a role for this chemotactic receptor in chronic TBI. In this context, CX3CR1 has been shown to have a neuroprotective role in chronic TBI, and CX3CR1 deficiency was recently shown to be associated with worse cognitive performance and increased neuronal cell death at day 30 after TBI [37, 38]. The decrease in Cx3cl1 (ligand) gene expression seen in our previous NanoString study and the upregulation of CX3CR1 seen in this study, raises the possibility that the stimulation of CX3CR1 by administration of exogenous CX3CL1 may further improve outcomes in chronic TBI.

With regard to CR4, published data, albeit scant, have shown different and often contrasting roles for this receptor in neurodevelopment, neuroinflammation and neurodegeneration. Specifically, CR4+ microglia along the white matter tracts were found to make up 20% of microglia in the early postnatal brain and to secrete insulin-growth factor 1 (Igf1) to support myelogenesis, astrocyte and neuronal differentiation, and matrix remodeling [39]. Conversely, in the adult brain in the context of experimental autoimmune encephalomyelitis, co-expression of CR4+ and MHC-II on microglia enhanced expression of co-stimulatory molecules such as CD80 and CD86, ultimately leading to higher proliferation of myelin oligodendrocyte glycoprotein-specific CD4+ T cells in vitro. However, compared to dendritic cells, which is the cell type expressing the highest levels of CR4, CD11c+ microglia did not express detectable levels of proinflammatory interleukins 6, 12 and 23 and induced lower levels of cytokines needed for the activation of T helper cells, namely Th1, Th17 and Th2, suggesting that they may display a reduced pro-inflammatory phenotype [40]. In the APP/PS1 mouse model of Alzheimer’s disease, the number of CR4 transcripts and CR4-positive microglia increased sharply and in parallel to the increasing Aβ plaque load with age, and their transcriptional profile showed an increase in immunosuppressive markers [41]. CR4 expression has also recently been described in association with stroke. Specifically, CR4+ microglia emerging one week post injury in the thalamus of mice exposed to ischemic stroke showed increased expression of several phagocytic markers similar to that described in aged APP/PS1 mice [42].

It is currently unclear whether the function of CR4 in general and of CR4+ microglia specifically is similar in different neuroinflammatory conditions, but there is consistency in the literature showing that microglia can phagocytose C3-opsonized synapses via complement receptor 3 (CR3). Considering that CR3 and CR4 bind the same complement ligands, it is tempting to speculate that these receptors may exert overlapping functions. There are, nevertheless, also nonoverlapping ligands for the two receptors [43]. For example, CR4 is dominant in adhesion to fibrinogen [44], and CR3 is inclined to bind positively charged species, whereas CR4 binds negatively charged species [43], and their intracellular tails differ markedly, suggesting distinctive functions [45]. Here, we found that at 28 days post-TBI, virtually all immune cell types in the brain up-regulated CR4, especially a subpopulation of microglia. However, we also found no effect for CR2-Crry on CR4 expression on microglia despite improved recovery. While this may at first suggest that the therapeutic effect of CR2-Crry is independent of CR4 levels on microglia, it may also be due to decreased pathologic interactions between CR4 and membrane-bound complement opsonins or due to protective CR4 effects that are more pronounced with decreased CR3-mediated synaptic uptake and complement activation. This widespread expression of CR4 on immune cells, which may potentially exert different and even opposing functions, currently make it difficult to speculate on the role of CR4 in neuroinflammation and neurodegeneration following TBI in the absence of CR4-targeted studies.

In this this study we also showed significant upregulation of C5aR1 on many immune cell types at days 3 & 7 after TBI. These findings are consistent with our previous transcriptomic findings in the same TBI model [29]. C5aR1 is a G-protein coupled receptor (GPCR) coupled to Gαi and upon binding to C5a leads to calcium influx, activation of phosphatidylinositol 3-kinase (PI3K) and mitogen-activated protein kinases (MAPKs), release of IL-6 cytokine, and immune cell migration [32]. The binding of C5aR1 to C5a also activates βarr1/2 which is responsible for the internalization of C5aR1. C5aR1 is primarily (although not exclusively) expressed on immune cells, and as such, has been implicated in proinflammatory signaling in numerous disorders including ischemic stroke and spinal cord injury. While pharmacological or genetic inhibition of C5aR1 in stroke and spinal cord injury has shown neuroprotective effects in several studies, here we showed that inhibition of C5aR1 did not ameliorate tissue loss or neurological deficits in TBI, despite decreasing immune cell counts in the injured brain [46–48]. This suggests that the neuroprotective effects observed with the C3 inhibitor CR2-Crry are likely mediated by blocking the generation of complement activation products upstream of C5a. Our previous data ruled out a role for the membrane attack complex, the terminal complement activation product, in 28-day outcomes after TBI using the same CCI model [49]. On the other hand, previous data from our lab in chronic TBI showed deposition of C3 opsonins on viable neuronal cells and synapses, together with C3-dependent microglia phagocytosis of synapses. These previous findings indicated a pathological role for an aberrant microglial-neuronal interaction, all of which is consistent with the findings reported here [7].

We did not investigate a dose–effect relationship between PMX205 dose and histological and behavioral outcomes and therefore cannot rule out a neuroprotective effect at higher doses of PMX205, although the same dosing strategy was protective in a murine model of Alzheimer’s disease [36] and it did affect immune cell recruitment in our model. Nevertheless, even though our data does not support a prominent role C5aR1 alone in shaping TBI outcomes, it does not exclude a role for C5aR1 in TBI that is contingent on other effector pathways. For example, in the presence of MAC activation, the inhibition of C5aR1-mediated immune cell activation may prevent the clearance of cellular debris from MAC-induced cell death and cause chronic accumulation of cellular toxins. As such, the neuroprotective effects of CR2-Crry may also be contingent on the synergistic effect of inhibiting multiple complement effector pathways simultaneously. Moreover, C5aR1 has been shown to promote the pluripotency and proliferation of neural progenitor cells in mice, suggesting that C5aR1 may also be involved in neuronal repair and regeneration in the adult brain after injury [50]. For instance, one study showed increased expression of C5aR1 after SCI, which was shown histologically to primarily colocalize with neurons and astrocytes [51]. Nevertheless, the specific role of non-immune expression of C5aR1 in the adult brain remains unexplored in either the normal or injured brain. Finally, C5a also binds to C5aR2, which has been shown to have similar pattern of immune cellular expression as C5aR1 [52]. However, in our study, unlike C5aR1, C5aR2 shows no significant change in the level expression on most immune cell types after injury (Additional file 1: Fig. S2). Since PMX205 is a specific antagonist to C5aR1, future studies targeting C5aR2 are needed to explore the role of C5aR2 in immune cell recruitment in TBI.

## Conclusion

This study implicates several immune cell types in the pathogenesis of TBI and highlights a potentially important role for CR4 and CR4+ microglia in chronic TBI. Moreover, this study showed that complement C5aR1 is involved in the infiltration of peripheral immune cells but does not alone significantly influence TBI outcomes. Our findings, together with previous data ruling out a role for the MAC in this model, suggest that the neuroprotective effects of complement inhibition are mediated by complement activation products upstream of C5 cleavage, especially C3 opsonins, although a combination of complement effector mechanisms may be operative. Our data point to future studies investigating the role of CR4 in TBI and other neurodegenerative disease, for which conditional knock out mice may be particularly useful.

## Supplementary Information

**Additional file 1: Fig. S1.** Flow SOM clustering and merging of immune cells based on lineage markers. **a** Heatmap showing the median scaled expression of 20 markers by each of the 40 lineage clusters returned by Flow SOM algorithm. **b** UMAP of the 40 clusters. **c** Similar heatmap for the 13 immune cell types resulting from the manual merge of the 40 lineage clusters. **Fig. S2.** Expression of functional receptors by immune cell types after TBI. Summary of the fold change in the expression of the 26 functional receptors by each of the 13 immune cell types at days 3, 7 and 28 after TBI compared to the sham group. Data are represented as the mean and are color coded for statistical significance. Blue color = decreased expression after injury. Warm color = increased expression after injury. Gray = no significant change in expression. False discovery rate was used to adjust the p-value for multiple comparisons. N = 6 per group. **Fig. S3.** Functional clusters based on the expression of functional receptors. **a** Heatmap of the 40 functional clusters returned by Flow SOM clustering of the cells based on the expression of 26 functional receptors, including complement, scavenger, and purinergic receptors. The median scaled expression of the functional receptors is shown for each cluster. **b** Boxplot showing the percentage of each functional cluster in PBS-treated groups compared to the sham group. Significant changes are colored red, blue and purple for days 3, 7 and 28 after TBI respectively. Non-significant changes are in gray. False discovery rate was used to adjust the p-value for multiple comparisons. N = 6 per group. **Fig. S4.** Effect of CR2-Crry on the percentage of the 40 functional clusters. **a** Boxplot showing the percentage of each functional cluster in PBS-treated groups vs CR2-Crry treated groups. Significant changes are summarized in figure 5e. N = 6 per group. **Table S1.** List of 14 barcodes and their respective samples in a batch.
