# Supplementary material for: Mass cytometric analysis of the immune cell landscape after traumatic brain injury elucidates the role of complement and complement receptors in neurologic outcomes
Source: Acta Neuropathol Commun. 2023 Jun 12;11:92. doi: 10.1186/s40478-023-01583-0 (PMC10258994; doi:10.1186/s40478-023-01583-0)
Supplement: Supplementary file 1 — Additional file 1: Fig. S1. Flow SOM clustering and merging of immune cells based on lineage markers. a Heatmap showing the median scaled expression of 20 markers by each of the 40 lineage clusters returned by Flow SOM algorithm. b UMAP of the 40 clusters. c Similar heatmap for the 13 immune cell types resulting from the manual merge of the 40 lineage clusters. Fig. S2. Expression of functional receptors by immune cell types after TBI. Summary of the fold change in the expression of the 26 functional receptors by each of the 13 immune cell types at days 3, 7 and 28 after TBI compared to the sham group. Data are represented as the mean and are color coded for statistical significance. Blue color = decreased expression after injury. Warm color = increased expression after injury. Gray = no significant change in expression. False discovery rate was used to adjust the p-value for multiple comparisons. N = 6 per group. Fig. S3. Functional clusters based on the expression of functional receptors. a Heatmap of the 40 functional clusters returned by Flow SOM clustering of the cells based on the expression of 26 functional receptors, including complement, scavenger, and purinergic receptors. The median scaled expression of the functional receptors is shown for each cluster. b Boxplot showing the percentage of each functional cluster in PBS-treated groups compared to the sham group. Significant changes are colored red, blue and purple for days 3, 7 and 28 after TBI respectively. Non-significant changes are in gray. False discovery rate was used to adjust the p-value for multiple comparisons. N = 6 per group. Fig. S4. Effect of CR2-Crry on the percentage of the 40 functional clusters. a Boxplot showing the percentage of each functional cluster in PBS-treated groups vs CR2-Crry treated groups. Significant changes are summarized in figure 5e. N = 6 per group. Table S1. List of 14 barcodes and their respective samples in a batch. [file 40478_2023_1583_MOESM1_ESM.pdf]

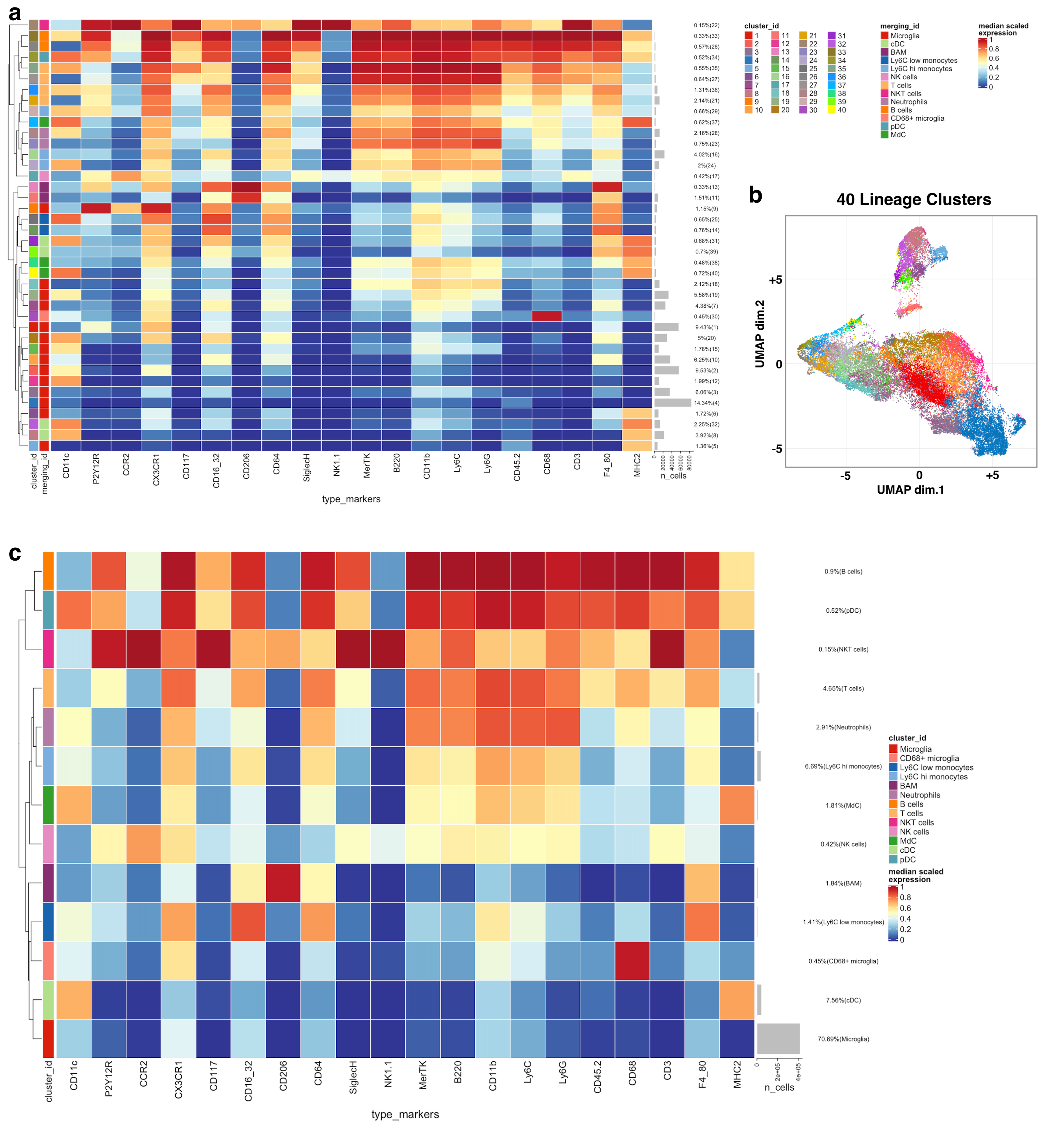


**Figure S1. FlowSOM clustering and merging of immune cells based on lineage markers.**
**a** Heatmap showing the median scaled expression of 20 markers (16 lineage and 4 functional) by each of the 40 lineage clusters returned by FlowSOM algorithm. **b** UMAP of the 40 clusters. **c** Similar heatmap for the 13 immune cell types resulting from the manual merge of the 40 lineage clusters.

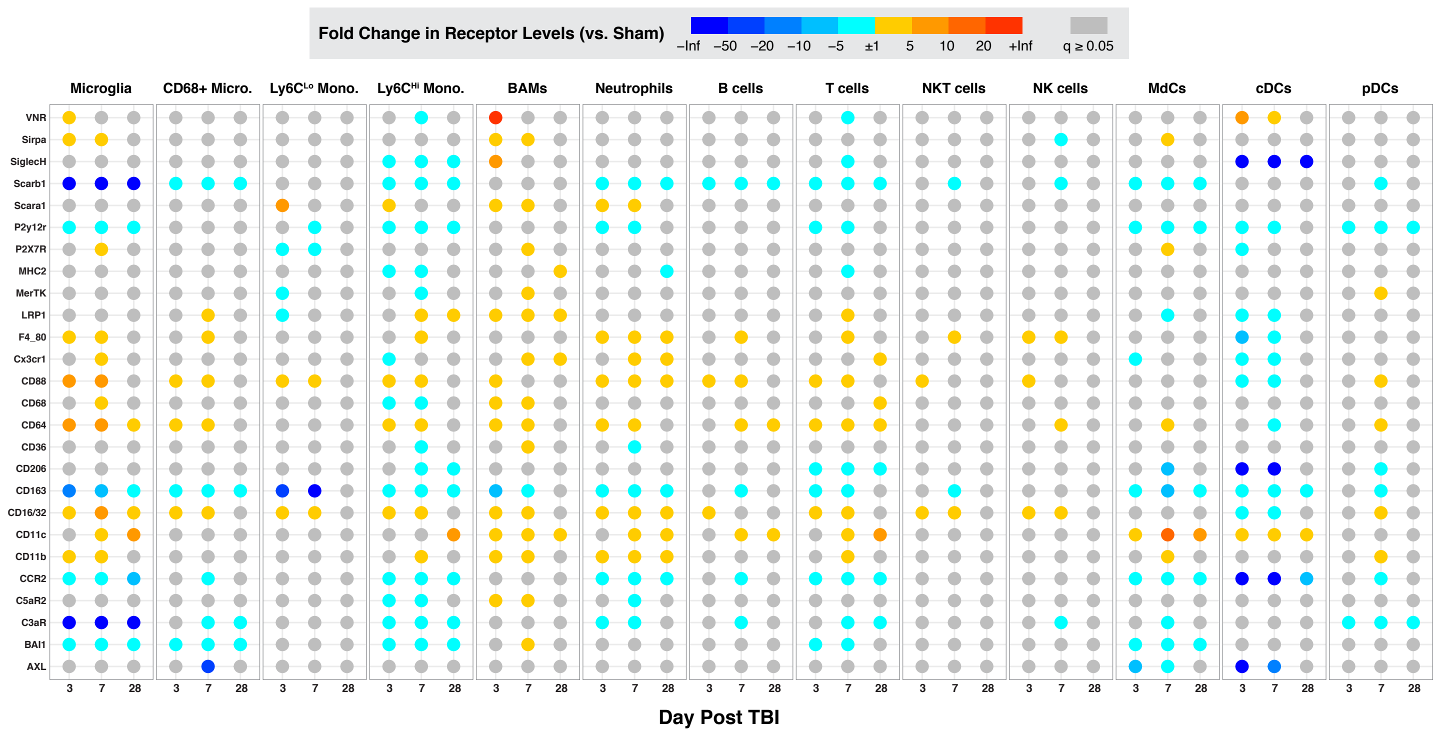


**Figure S2. Expression of functional receptors by immune cell types after TBI.**
Summary of the fold change in the expression of the 26 functional receptors by each of the 13 immune cell types at days 3, 7 and 28 after TBI compared to the sham group. Data are represented as the mean and are color-coded for statistical significance. Blue color = decreased expression after injury. Warm color = increased expression after injury. Gray = no significant change in expression. False discovery rate was used to adjust the p-value for multiple comparisons. N = 6 per group.

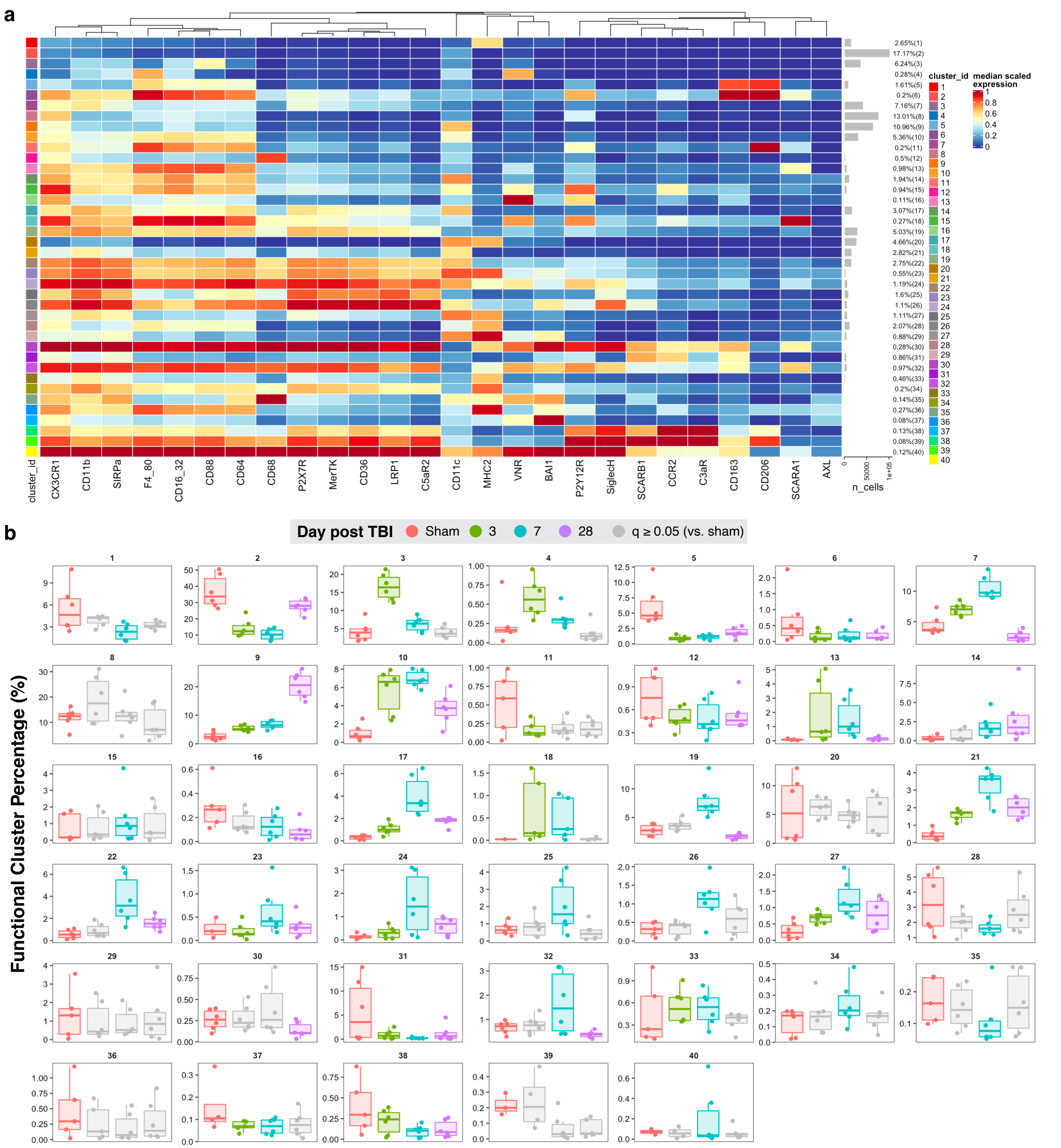


**Figure S3. Functional clusters based on the expression of functional receptors.**

**a** Heatmap of the 40 functional clusters returned by FlowSOM clustering of the cells based on the expression of 26 functional receptors, including complement, scavenger, and purinergic receptors. The median scaled expression of the functional receptors is shown for each cluster. **b** Boxplot showing the percentage of each functional cluster in PBS-treated groups compared to the sham group. Significant changes are colored red, blue and purple for days 3, 7 and 28 after TBI respectively. Non-significant changes (q ≥ 0.05) are in gray. False discovery rate was used to adjust the p-value for multiple comparisons. N = 6 per group.

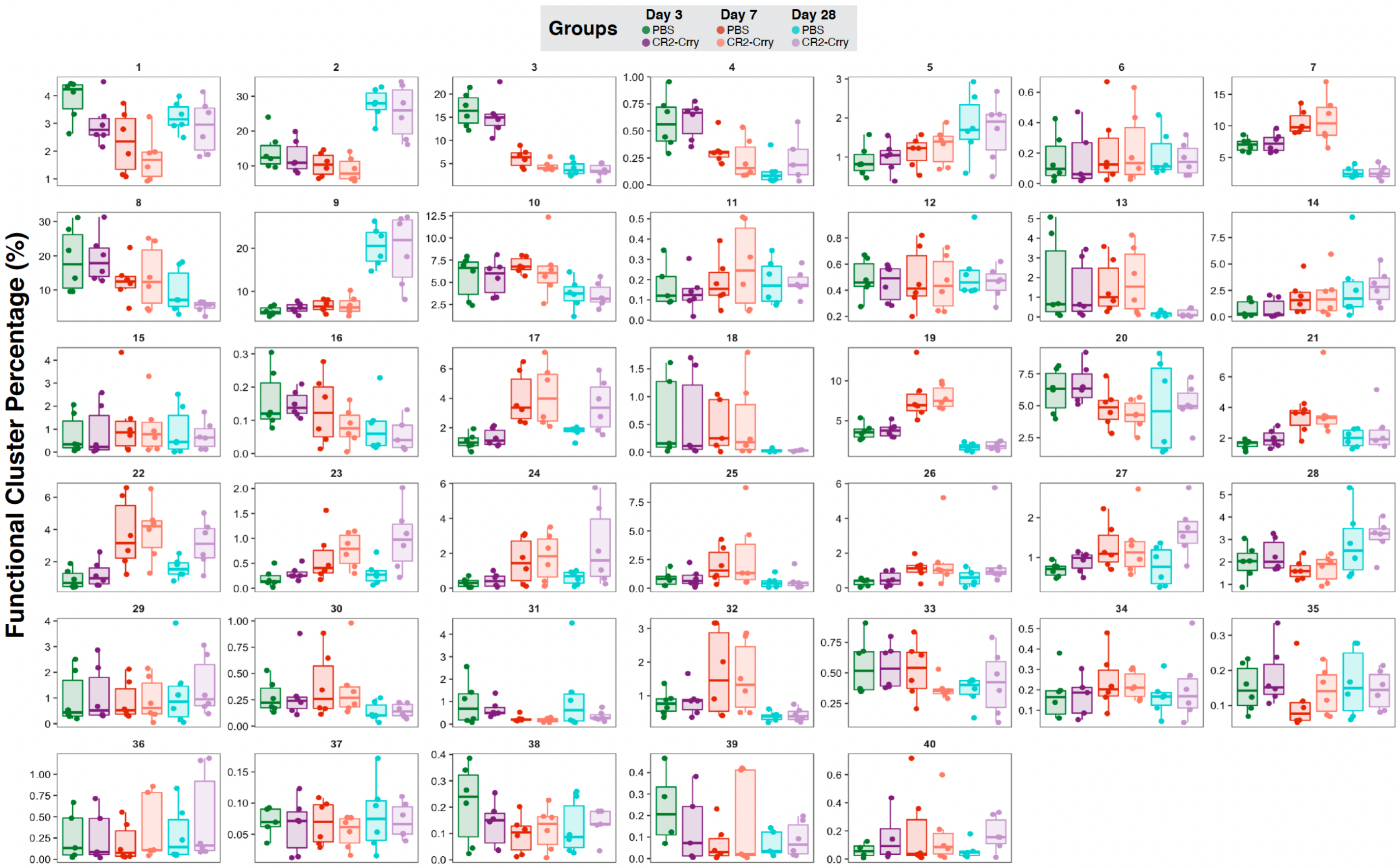


**Figure S4. Effect of CR2-Crry on the percentage of the 40 functional clusters.**
**a** Boxplot showing the percentage of each functional cluster in PBS-treated groups vs CR2-Crry treated groups. Significant changes are summarized in figure 5e. N = 6 per group.

**Table S1. List of 14 barcodes and their respective samples in a batch.**

| Samples Per Batch | Heavy metal + isotope | | | | | |
|---|---|---|---|---|---|---|
| | In113 | In115 | Pt194 | Pt195 | Pt196 | Pd105 |
| **1** | 1 | 1 | 1 | 0 | 0 | 0 |
| **2** | 1 | 1 | 0 | 1 | 0 | 0 |
| **3** | 1 | 1 | 0 | 0 | 1 | 0 |
| **4** | 1 | 1 | 0 | 0 | 0 | 1 |
| **5** | 1 | 0 | 1 | 1 | 0 | 0 |
| **6** | 1 | 0 | 1 | 0 | 1 | 0 |
| **7** | 1 | 0 | 1 | 0 | 0 | 1 |
| **8** | 1 | 0 | 0 | 1 | 1 | 0 |
| **9** | 1 | 0 | 0 | 1 | 0 | 1 |
| **10** | 1 | 0 | 0 | 0 | 1 | 1 |
| **11** | 0 | 1 | 1 | 1 | 0 | 0 |
| **12** | 0 | 1 | 1 | 0 | 1 | 0 |
| **13** | 0 | 1 | 1 | 0 | 0 | 1 |
| **14** | 0 | 1 | 0 | 1 | 1 | 0 |

**Legend.** Data was acquired from a total of 42 samples across 3 batches, each containing 14 samples labeled with specific barcodes as in the table. All cells were de-barcoded and assigned to their sample prior to data analysis using the CATALYST R package.
